# Discrete choice experiment data for street-level urban greening in Berlin

**DOI:** 10.1016/j.dib.2019.105027

**Published:** 2019-12-18

**Authors:** Erik Fruth, Michele Kvistad, Joe Marshall, Lena Pfeifer, Luisa Rau, Julian Sagebiel, Daniel Soto, John Tarpey, Jessica Weir, Bradyn Winiarski

**Affiliations:** Technische Universität Berlin, Germany

**Keywords:** Discrete choice experiment, Urban green, Urban ecosystem services, Willingness to pay, Green facades, Street greening, Environmental initiatives, Berlin

## Abstract

The data presented in this DiB article are the outcome of a survey implemented in a Berlin neighborhood from January to March 2018. The data consist of socio-demographic, attitudinal and perception questions, and, most importantly, a discrete choice experiment. This dataset is complementary to the full research article, “Economic valuation of street-level urban greening: A case study from an evolving mixed-use area in Berlin” [1]. The analysis of the discrete choice experiment provided in the full article could be used to guide policy- and project-level decision-making for green building practices and urban green initiatives, while the dataset available here can be used to provide insight about how our sample population responded to the remaining parts of the questionnaire and how the experiment could be replicated in context or elsewhere in Berlin.

**Table:** Specifications Table

Subject	Management, Monitoring, Policy and Law
Specific subject area	Environmental valuation using a Stated Preference study (Discrete Choice Experiment) to quantify users' Willingness to Pay for urban green features
Type of data	Table
How data were acquired	Electronic discrete choice experiment survey Instrument: SurveyEngine online platform
Data format	Raw: .csv Filtered: .csv
Parameters for data collection	Survey respondents were limited to ‘frequent users’ of the area, where the survey took place (i.e. self-reported residents, workers, or users) over 18 years old. The online survey was active from January to March 2018, limiting the responses to those months. Due to the survey's online aspect, a mobile device or computer was required to complete the survey in either German or English language.
Description of data collection	To maximize awareness of our web-based survey, we canvassed local businesses and placed flyers in mailboxes on selected streets within the sampling area with information about the survey's background, purpose, and its weblink. We posted the survey's URL on social media platforms and in local newsletters with a short description. All data provided here were collected electronically from the online survey responses inputted into the SurveyEngine platform. The data is not representative of the target population and the data collection process was not random.
Data source location	Potsdamer Straße neighborhood, Berlin Germany
Data accessibility	All data are on a public repository. Repository name: Mendeley Data identification number: 10.17632/sjgr3w28n7.2 Direct URL to data: https://data.mendeley.com/datasets/sjgr3w28n7/2
Related research article	Erik Fruth, Michele Kvistad, Joe Marshall, Lena Pfeifer, Luisa Rau, Julian Sagebiel, Daniel Soto, John Tarpey, Jessica Weir, Bradyn Winiarski Economic valuation of street-level urban greening: A case study from an evolving mixed-use area in Berlin Land Use Policy, https://doi.org/10.1016/j.landusepol.2019.104237

**Table:** 

**Value of the Data** • The dataset provides the basis for a novel investigation of preferences related to street-level greening measures in Berlin. • The data can be used by city-planners and policy-makers to understand the preferences for (and general interest in) urban green features in Berlin. • The data can be used as a baseline/supplement for further analyses about larger-scale interventions, the nuances of preference differences, and the effects of sociodemographic variables on those differences, or could be included in other valuation studies, e.g. cost benefit analyses.

## 1 Data

### 1.1 Raw

“Deutsch_potsdamer_experiment_15” = .csv file, contains the raw choice data from the DCE portion of the German-language survey; used in the logistic regression analysis to establish WTP.

“English_postdamer_experiment_15” = .csv file, contains the raw choice data from the DCE portion of the English-language survey; used in the logistic regression analysis to establish WTP.

“table_2019-07-27_11-28-18” = .csv file with age/gender/population figures from StatIS-BBB [2]; source file for the Berlin (parent population) age/gender/population data points in our sociodemographic analysis.

“SB_O02-03-00_2013j05_BE” = .csv file with household income data [3]; source file for the Berlin (parent population) income data points in our sociodemographic analysis.

### 1.2 Filtered

“Deutsch_potsdamer_covariates” = .csv file, IP address and user comments have been removed to protect respondents' confidentiality; used in our sociodemographic analysis.

“English_potsdamer_covariates” = .csv file, IP address and user comments have been removed to protect respondents' confidentiality; used in our sociodemographic analysis.

## 2 Experimental design, materials, and methods

The economic valuation of urban green is elicited through a discrete choice experiment (DCE), described in a complementary full research article, “Economic valuation of street-level urban greening: A case study from an evolving mixed-use area in Berlin” [1]. DCEs are questionnaire-based, providing respondents with a hypothetical market in which they can choose to “buy” the good or not. The good is characterized by several attributes that vary in predefined levels according to an experimental design – in this case, an orthogonal array. The respondents choose between presented alternatives with varying attribute levels several times. The generated data consist of choices made by the respondents (the dependent variable) and attributes and socio-demographic characteristics (independent or explanatory variables). Information about the foundations and applications of DCEs as well as how to analyze the resulting datasets in the statistical computing software R is provided in Aizaki et al. [4].

The selection of attributes was informed by initial field research: consultations and feedback from an established local interest group associated with the research project, Boulevard Potsdamer, site visits to the sampling area, and expert interviews with municipal district offices and local environmental organizations. The three selected attributes presented our respondents with tangible possibilities and were differentiated to facilitate realistic combinations: 1) the number of green building facades, 2) street green in the form of trees or planter boxes, and 3) green initiatives like eco-events and education programs. The attributes, which included detailed descriptions and visual representations, were pre-tested on a selection of students and scientists at the Technische Universität Berlin and also with a convenience sample of about 20 participants.

Each attribute consisted of three levels: the present condition (status quo) in the sampling area and two higher levels that represented improvement to the attribute. To maintain transparency and assure a common interpretation of our survey, each of the attributes included detailed descriptions that explained their potential positive and negative effects on the neighborhood. Additionally, a cost attribute, presented as a mandatory annual payment from individuals to an “urban green fund” managed by the local government, was included in order to calculate Willingness to Pay (WTP). The cost consisted of six levels ranging from 12 € to 360 €, with the exception of the status quo which remained at 0 €. The cost served as a proxy to establish respondents' Willingness to Pay and did not represent actual implementation costs for the selected attributes.

The design of the questionnaire consisted of five sections. In the first section, respondents were asked about their use of Potsdamer Straβe, including mode of transportation, and their understanding of two environmental terms (“ecosystem services” and “biodiversity”) that appeared later in the attribute descriptions. A screening question identified respondents not living in, working in, or frequently using the sampling area (i.e. not part of our target population) and directed them to the end of the survey. In the second section, respondents were asked to imagine a hypothetical situation where their local district offices would use an “urban green fund” for Berlin to develop more green features on the selected section of Potsdamer Straβe. In the third section, the choice experiment was explained; the attributes were defined with photos and descriptions of the levels of improvement for each attribute, and the status quo was presented for each respective attribute. The fourth section was the discrete choice experiment itself, where respondents were provided nine randomly selected choice sets from an orthogonal array of 18. Within the choice sets a mouse-over option allowed the respondents to review the descriptions of the attribute levels. Each choice set presented respondents with three alternatives where they must choose the preferred alternative based on the levels of the attributes and the required monetary contribution. This end of this section included a question on respondents' anticipated participation in eco-events and educational programming if the urban green measures were implemented. Socio-demographic questions on age, gender, employment status, and income, each with the option of “prefer not to state,” concluded the final section of the survey questionnaire.

SurveyEngine, a market research company focused on choice modelling research, administered the discrete choice experiment. In order to increase accessibility and inclusivity, respondents were able access the survey at their convenience via either desktop or mobile devices from January 2018 until March 2018. Informational materials containing a brief description and the online survey link were distributed to residents and local businesses in the form of flyers placed in residential mailboxes and business cards and flyers distributed to local businesses and residents within the sampling area. Additionally, Boulevard Potsdamer distributed the survey weblink via their mailing list, newsletter, and social media. Respondent participation was incentivized by offering a voucher at the end of the survey to be used at a local business. It should be noted that the sample is not random and self-selection and no-response bias could exist. It is not possible to use the data to extrapolate results to the entire sampling area.

The SurveyEngine platform produced two datasets for each language version of our experiment, resulting in a total of four datasets (“Deutsch_potsdamer_experiment_15” and “Deutsch_potsdamer_covariates”; “English_potsdamer_experiment_15” and “English_potsdamer_covariates”), which are provided alongside this article.
