# Supplementary material for: Discrete choice experiment data for street-level urban greening in Berlin
Source: Data Brief. 2019 Dec 18;28:105027. doi: 10.1016/j.dib.2019.105027 (PMC6956670; doi:10.1016/j.dib.2019.105027)
Supplement: Multimedia component 2 [file mmc2.pdf]

Potsdamer Str. Survey - Google Chrome

Secure | https://b1.surveyengine.com/bin/play.pl?PLAYSID=cd49dcb0d9baf7f15d0a16e0c017b20d&page_number=1

1. Introducti...

Potsdamer Str. Survey

Hello and thank you for participating in our survey!

We are a group of Environmental Planning Master's students from the Technische Universität Berlin conducting research in collaboration with Boulevard Potsdamer on the urban green features in the neighbourhood around Potsdamer Straße. The analysis will focus on prioritising greening measures in the area to enhance the services provided by environmental features for the health, well-being and recreation of citizens. Some examples of urban greening measures include: urban gardens, green building facades, trees and plants on the street, and environmental education events and programs. The study focuses on the preferences of people who frequent the area around Potsdamer Straße.

The survey should take only 10 minutes of your time, it is voluntary and your responses will be completely anonymous. Responses to this survey cannot be traced back to the respondent. No personally identifiable information is captured unless you voluntarily offer personal or contact information in any of the comment fields. If you have any questions, please feel free to email us at urbangreenberlin@gmail.com.

As a thank you for your participation, we would like to offer you a voucher for a FREE hot beverage, redeemable at the Nachbarschaftstreff Cafe at the Bibliothek Tiergarten-Süd, Lützowstraße 27. Please write down, screenshot or print the final page of the survey where you see your unique voucher number and stop by the library. It is valid until February 28th, 2018.

Thank you for your input, we hope you find the survey insightful!

Technische Universität Berlin

BOULEVARD POTSDAMER
Eine Straße wird GRÜN!

prev | next

© 2017 SurveyEngine P/L. Version 3.1

Potsdamer Str. Survey - Google Chrome

Secure | https://b1.surveyengine.com/bin/play.pl?PLAYSID=cd49dcb0d9baf7f15d0a16e0c017b20d&page_number=1

2. Getting to know...

Potsdamer Str. Survey

How do you use Potsdamer Straße?

Select all that apply

- ☐ I live in the neighbourhood
- ☐ I work in the neighbourhood
- ☐ I frequent the area (children attend school/kindergarten, go shopping or to lunch here, etc.)
- ☐ None of the above

How do you commute along Potsdamer Straße?

Select one response from each row

	Never	Often	Always
Walk	○	○	○
Bike	○	○	○
Public Transit	○	○	○
Car or Carpool	○	○	○
Other, i.e. scooter/moped	○	○	○

Are you satisfied with the current number of trash bins and recycling bins found along Potsdamer Straße?

Select only one answer

- Very satisfied
- Satisfied
- Somewhat satisfied
- Not satisfied
- Not sure

Are you aware of the term ecosystem services?

Select only one answer

- Yes
- No

Ecosystem services (ESS) are defined as benefits which humans gain from ecosystems. Examples of ecosystem services include food and water production, pollination, reducing air pollution and recreation or well-being.

Are you aware of the term biodiversity?

Select only one answer

- Yes
- No

Biodiversity is the variety of living things in a specific habitat. A very high level of biodiversity not only increases the number of potential ecosystem services provided, but it is also considered important to make ecosystems more resilient to stresses like diseases or climate change.

prev next

© 2017 SurveyEngine P/L. Version 3.1

OPT-OUT for NON-USERS:

Potsdamer Str. Survey - Google Chrome

Secure | https://b1.surveyengine.com/bin/play.pl?PLAYSID=4b8ce3ddc1ad70e938718bf305e88591&page_number=1

19. Quicke...

Potsdamer Str. Survey

Unfortunately, you are not eligible to participate in the survey. Only people that live in, work in and/or use Potsdamer Str. frequently can participate in the survey. Still, we very much appreciate your time and willingness to participate. If you would like further information about this survey and the research being done by the Technische Universität Berlin, you can email us at: urbangreenberlin@gmail.com Thank You!

prev submit answers and finish

© 2017 SurveyEngine P/L. Version 3.1

Potsdamer Str. Survey - Google Chrome

Secure | https://b1.surveyengine.com/bin/play.pl?PLAYSID=cd49dcb0d9baf7f15d0a16e0c017b20d&page_number=1

3. About the st...

Potsdamer Str. Survey

Consider the following situation: The district office (Bezirksamt) for Potsdamer Straße neighborhood has interest to develop more green features in the area. The cost of the new greening measures would be paid for by citizens through a yearly mandatory contribution to a fund for urban green in Berlin. This fund is managed by the Bezirksamt, which is the city's administrative body responsible for green infrastructure .

The area of focus is a 250 m zone around the main stretch of Potsdamer Straße south of the Landwehr canal. See map below for reference.

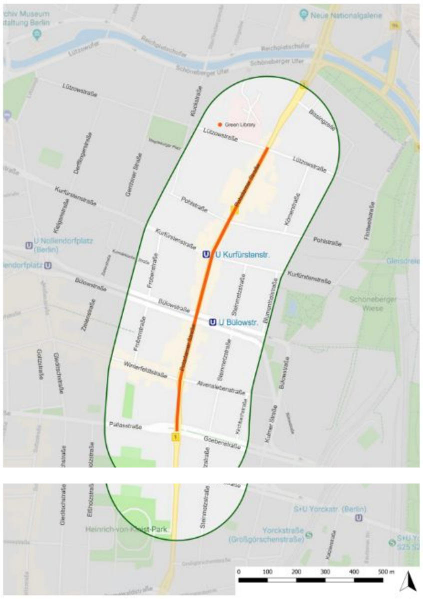

Please carefully read the information in the next sections about the green features being proposed.

prev

next

© 2017 SurveyEngine P/L. Version 3.1

Potsdamer Str. Survey - Google Chrome

Secure | https://b1.surveyengine.com/bin/play.pl?PLAYSID=cd49dcb0d9baf7f15d0a16e0c017b20d&page_number=1

4. Green building f...

Potsdamer Str. Survey

Green feature1: **Street-facing green facades**

Green facades are the outward walls of a building covered by vegetation. They are created by placing plant-containing structures against the building or by growing climbing plants directly onto the building itself. They can provide an attractive look to the wall, although this can change seasonally. The plants can provide ecosystem services like insulating buildings against heat and cold, reducing air pollution, increasing biodiversity and reducing street noise levels. However, green facades can also damage walls, involve maintenance and attract unwanted wildlife.

Currently the area of interest has only a few (less than 1 in 10) street-facing green facades (Status quo).
Street-facing green facades are proposed to be increased to one of the following levels:

- 1 out of 10 buildings with green facades.
- 2 out of 10 buildings with green facades.

Here are some examples of what green facades might look like on a building:

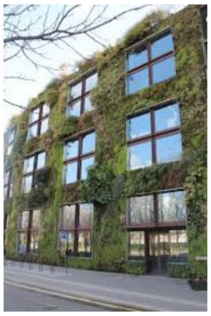

Photo: PxHere

Photo: PxHere

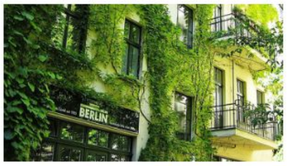

Photo: Pixa Bay

prev

next

© 2017 SurveyEngine P/L. Version 3.1

Green feature 2: **Street greening**

Street greening is the addition of trees, sidewalk gardens (planters) and natural vegetation along Potsdamer Straße. Street greening measures are controlled by infrastructure planning and play a significant role in providing ecosystem services to communities such as creating shade, improving street-level air quality, lowering air temperature, absorbing noises and reducing wind speed in streets. However, it can also obstruct footpaths and attract unwanted wildlife, as well as cause inconveniences as a result of fallen leaves or fruits.

Currently the study area has on average 1 tree every 20m, no maintained vegetation and a few small planters (Status quo).
Street greening are proposed to be increased to the following levels:

- Mostly trees - Trees every 10m, few planters.
- Mostly vegetation - Trees every 20m, many planters.

Here are some examples of what street greening might look like:

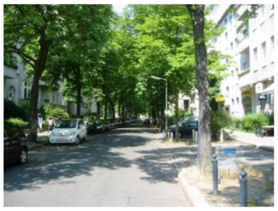

Photo: Wikimedia Commons

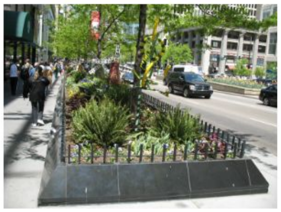

Photo: Wikimedia Commons

© 2017 SurveyEngine P/L. Version 3.1

Green feature 3: **Green initiatives**

Green initiatives create opportunities for businesses and the community to learn about environmentally-friendly behaviors in a recreational setting. These projects and programs encourage the street to "go green" while stimulating the economy and creating a sense of social cohesion. However, large events can also create unwanted noise, congestion of streets and increase demand for street maintenance. Green initiatives can be broken down into two main categories: 1) Eco-events and 2) Educational programming.

- *Eco-events* are any free community-wide events which are considered environmentally focused. Potential events could include "Potsdamer Straße Green Week," a mass tree planting, or competitions to create "green" balconies or artworks.
- *Educational programming* is described as a variety of workshops and small group activities for all ages designed to inform participants about the ecosystem services provided by urban green. Examples include: waste management programs for businesses and residences, urban gardening classes, environmental art/upcycling classes, workshops about sustainable foods, energy efficiency, environmental design and architecture, etc.

The community currently has irregular programs and events throughout the year (Status quo).
Green initiatives are proposed to be introduced at the following levels:

- Occasionally - Eco-events once per year, monthly educational programming.
- Often - Eco-events once per season, weekly educational programming.

Here are some examples of what green initiatives might look like:

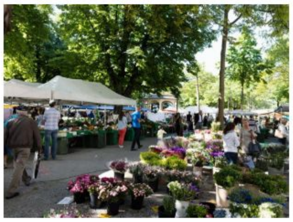

Photo: zuerich.com

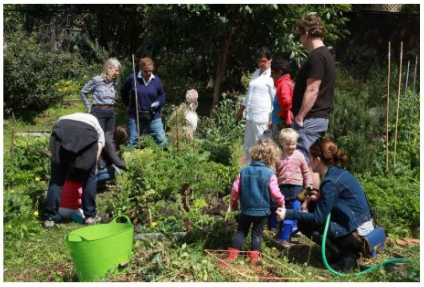

Photo: Curbed

prev   next

© 2017 SurveyEngine P/L. Version 3.1

Potsdamer Str. Survey - Google Chrome

Secure | https://b1.surveyengine.com/bin/play.pl?PLAYSID=cd49dcb0d9baf7f15d0a16e0c017b20d&page_number=1

7. Experiment ...

Potsdamer Str. Survey

For the following questions, we ask you to consider all terms and information provided in the previous sections about the green features.

Consider now that the Bezirksamt is trying to identify which of these measures are in line with the preferences of the people who frequently use this street. Imagine that you could participate in the decision process.

You will be shown a series of 9 choices with different combinations of different levels of greening features as well as a yearly contribution amount. Please select your preferred option when considering how much to contribute to the potential new fund, Urban Green in Berlin. Consider what you are willing and able to pay for the greening features around Potsdamer Straße. If you are not interested, or if you are not willing to pay for such measures, you can also choose the status quo - which means that no changes would occur as well as no payment.

	Option 1	Option 2	Status Quo
**Green Facades**	None	None	None
**Street Greening**	Mostly planters	Few trees and planters	Few trees and planters
**Green Initiatives**	Occasionally	Occasionally	Irregular
**Yearly fee**	€120	€120	no additional costs
**Which would you choose?**	○	○	○

prev next

© 2017 SurveyEngine P/L. Version 3.1

Potsdamer Str. Survey - Google Chrome

Secure | https://b1.surveyengine.com/bin/play.pl?PLAYSID=cd49dcb0d9baf7f15d0a16e0c017b20d&page_number=1

8. Experiment ...

Potsdamer Str. Survey

Of the three options below, select your preferred option

	Option 1	Option 2	Status Quo
**Green Facades**	2 out of 10 buildings	None	None
**Street Greening**	Mostly planters	Few trees and planters	Few trees and planters
**Green Initiatives**	Often	Often	Irregular
**Yearly fee**	€30	€30	no additional costs
**Which would you choose?**	○	○	○

prev next

© 2017 SurveyEngine P/L. Version 3.1

Potsdamer Str. Survey - Google Chrome

Secure | https://b1.surveyengine.com/bin/play.pl?PLAYSID=cd49dcb0d9baf7f15d0a16e0c017b20d&page_number=1

9. Experiment ...

Potsdamer Str. Survey

Of the three options below, select your preferred option

	Option 1	Option 2	Status Quo
Green Facades	None	None	None
Street Greening	Mostly trees	Few trees and planters	Few trees and planters
Green Initiatives	Often	Often	Irregular
Yearly fee	€60	€60	no additional costs
Which would you choose?			

prev next

© 2017 SurveyEngine P/L. Version 3.1

Potsdamer Str. Survey - Google Chrome

Secure | https://b1.surveyengine.com/bin/play.pl?PLAYSID=cd49dcb0d9baf7f15d0a16e0c017b20d&page_number=1

10. Experiment ...

Potsdamer Str. Survey

Of the three options below, select your preferred option

	Option 1	Option 2	Status Quo
Green Facades	1 out of 10 buildings	None	None
Street Greening	Mostly planters	Few trees and planters	Few trees and planters
Green Initiatives	Often	Often	Irregular
Yearly fee	€12	€12	no additional costs
Which would you choose?			

prev next

© 2017 SurveyEngine P/L. Version 3.1

Potsdamer Str. Survey - Google Chrome

Secure | https://b1.surveyengine.com/bin/play.pl?PLAYSID=cd49dcb0d9baf7f15d0a16e0c017b20d&page_number=1

11. Experiment ...

Potsdamer Str. Survey

Of the three options below, select your preferred option

	Option 1	Option 2	Status Quo
Green Facades	1 out of 10 buildings	None	None
Street Greening	Mostly trees	Few trees and planters	Few trees and planters
Green Initiatives	Occasionally	Occasionally	Irregular
Yearly fee	€30	€30	no additional costs
Which would you choose?			

prev

next

© 2017 SurveyEngine P/L. Version 3.1

Potsdamer Str. Survey - Google Chrome

Secure | https://b1.surveyengine.com/bin/play.pl?PLAYSID=cd49dcb0d9baf7f15d0a16e0c017b20d&page_number=1

12. Experiment ...

Potsdamer Str. Survey

Of the three options below, select your preferred option

	Option 1	Option 2	Status Quo
Green Facades	2 out of 10 buildings	None	None
Street Greening	Mostly trees	Few trees and planters	Few trees and planters
Green Initiatives	Often	Often	Irregular
Yearly fee	€360	€360	no additional costs
Which would you choose?			

prev

next

© 2017 SurveyEngine P/L. Version 3.1

Potsdamer Str. Survey - Google Chrome

Secure | https://b1.surveyengine.com/bin/play.pl?PLAYSID=cd49dcb0d9baf7f15d0a16e0c017b20d&page_number=1

13. Experiment ...

Potsdamer Str. Survey

Of the three options below, select your preferred option

	Option 1	Option 2	Status Quo
**Green Facades**	2 out of 10 buildings	None	None
**Street Greening**	Few trees and planters	Few trees and planters	Few trees and planters
**Green Initiatives**	Occasionally	Occasionally	Irregular
**Yearly fee**	€60	€60	no additional costs
**Which would you choose?**	○	○	○

prev next

© 2017 SurveyEngine P/L. Version 3.1

Potsdamer Str. Survey - Google Chrome

Secure | https://b1.surveyengine.com/bin/play.pl?PLAYSID=cd49dcb0d9baf7f15d0a16e0c017b20d&page_number=1

14. Experiment ...

Potsdamer Str. Survey

Of the three options below, select your preferred option

	Option 1	Option 2	Status Quo
**Green Facades**	None	None	None
**Street Greening**	Few trees and planters	Few trees and planters	Few trees and planters
**Green Initiatives**	Often	Often	Irregular
**Yearly fee**	€240	€240	no additional costs
**Which would you choose?**	○	○	○

prev next

© 2017 SurveyEngine P/L. Version 3.1

Potsdamer Str. Survey - Google Chrome

Secure | https://b1.surveyengine.com/bin/play.pl?PLAYSID=cd49dcb0d9baf7f15d0a16e0c017b20d&page_number=1

15. Experiment ...

Potsdamer Str. Survey

Of the three options below, select your preferred option

	Option 1	Option 2	Status Quo
**Green Facades**	None	None	None
**Street Greening**	Few trees and planters	Few trees and planters	Few trees and planters
**Green Initiatives**	Irregular	Irregular	Irregular
**Yearly fee**	€30	€30	no additional costs
**Which would you choose?**	○	○	○

prev next

© 2017 SurveyEngine P/L. Version 3.1

Potsdamer Str. Survey - Google Chrome

Secure | https://b1.surveyengine.com/bin/play.pl?PLAYSID=cd49dcb0d9baf7f15d0a16e0c017b20d&page_number=1

16. Stand-alone q...

Potsdamer Str. Survey

If green initiatives are set up, which activity would you participate in?

Select all that apply

- ☐ Eco-events
- ☐ Educational programming
- ☐ Would not participate

prev next

© 2017 SurveyEngine P/L. Version 3.1

Potsdamer Str. Survey - Google Chrome

Secure | https://b1.surveyengine.com/bin/play.pl?PLAYSID=cd49dcb0d9baf7f15d0a16e0c017b20d&page_number=1

17. Demograph...

Potsdamer Str. Survey

Age:

Select only one answer

- Under 18
- 18-24
- 25-34
- 35-44
- 45-54
- 55-64
- 65+
- Prefer not to state

The following questions are purely for statistical purposes.

Gender:

Select only one answer

- Female
- Male
- Other
- Prefer not to state

Employment status:

Select only one answer

- Full-time employment
- Part-time employment
- Not employed
- Student
- Retired
- Prefer not to state

Annual household income:

Select only one answer

- <€7,000
- €7,000–€15,000
- €15,000–€30,000
- €30,000–€45,000
- €45,000–€60,000
- >€60,000
- Prefer not to state

What other features do you think would help Potsdamer Straße become more green or eco-friendly?

Enter text below

prev

next

© 2017 SurveyEngine P/L. Version 3.1

Potsdamer Str. Survey - Google Chrome

Secure | https://b1.surveyengine.com/bin/play.pl?PLAYSID=cd49dcb0d9baf7f15d0a16e0c017b20d&page_number=1

18. Finish

Potsdamer Str. Survey

Click the submit button below to conclude the survey!

As a thank you for your time, we invite you to write down, screenshot, or print the voucher ID number below to redeem a FREE hot beverage at the Nachbarschaftstreff cafe at the "Green" Bibliothek Tiergarten-Süd (Lützowstraße 27)!

Your submissions will greatly help with the future development of greening measures along Potsdamer Straße. If you would like further information about this survey and the research being done by the Technische Universität Berlin, you can email us at: urbangreenberlin@gmail.com

Thank You!

Your Voucher ID: 245926

Final comments?

Enter text below

prev

submit answers and finish

© 2017 SurveyEngine P/L. Version 3.1
